# Nematicons and Their Electro-Optic Control: Light Localization and Signal Readdressing via Reorientation in Liquid Crystals

**DOI:** 10.3390/ijms141019932

**Published:** 2013-10-08

**Authors:** Armando Piccardi, Alessandro Alberucci, Gaetano Assanto

**Affiliations:** Nonlinear Optics and OptoElectronics Lab (NooEL), University of Rome “Roma Tre”, Via della Vasca Navale 84, Rome 00146, Italy; E-Mails: apiccardi@uniroma3.it (A.P.); alberucc@uniroma3.it (A.A.)

**Keywords:** reorientation, liquid crystals, nonlinear optics, nonlocality, solitons, self-focusing, beam steering

## Abstract

Liquid crystals in the nematic phase exhibit substantial reorientation when the molecules are driven by electric fields of any frequencies. Exploiting such a response at optical frequencies, self-focusing supports transverse localization of light and the propagation of self-confined beams and waveguides, namely “nematicons”. Nematicons can guide other light signals and interact with inhomogeneities and other beams. Moreover, they can be effectively deviated by using the electro-optic response of the medium, leading to several strategies for voltage-controlled reconfiguration of light-induced guided-wave circuits and signal readdressing. Hereby, we outline the main features of nematicons and review the outstanding progress achieved in the last twelve years on beam self-trapping and electro-optic readdressing.

## 1. Introduction

In the sixties, the invention of the laser made available optical fields of very high, formerly inaccessible, intensities, allowing the birth of nonlinear optics [1]. One of the most intriguing phenomena in nonlinear optics is the Kerr effect, that is, the dependence of the refractive index on the local optical intensity. For bell-shaped light beams, the Kerr effect is responsible for self-focusing: optical beams induce a lens-like refractive index distribution in the medium, in turn modifying their own width [2]. When nonlinear focusing balances beam diffraction in space, a shape and size-preserving nonlinear wave packet is formed, commonly called a (spatial) soliton. Solitons, as entities stemming counteracting linear and nonlinear responses, have been discovered in various branches of physics, from optics to fluidodynamics, from chemistry to solid-state physics [3].

Spatial optical solitons (hereafter, simply solitons) have been largely investigated since the inception of nonlinear optics [4], including numerous families, types and nonlinear mechanisms [5]. Due to their ability to guide other optical signals, a large portion of the current research on solitons aims at all-optical signal processing via the possibility of realizing/controlling photonic devices by light itself and in real time. Developing/improving strategies for the control of such self-induced waveguides is therefore crucial. In this scenario, nematic liquid crystals (NLC), *i.e*., liquid crystals in the nematic phase, were found to compete well with other nonlinear optical materials, including thermal [6,7], photorefractive [8] and quadratic media [9,10]. NLC exhibit large reorientational nonlinearity, extended spectral transparency, low dielectric constant, tunability of linear and nonlinear properties, large damage threshold and high nonlocality [11]. Microscopically, both the high reorientational response and the tunability originate from the ease of NLC molecules to rotate under the application of an electric field, regardless of its frequency: in the macroscopic scale, this yields a large polarization-dependent electro-optic effect [12] and a nonlinear Kerr-like response [13]. The remarkable reorientational nonlinearity allows the excitation of spatial solitons with low power CW (Continuous Wave) sources [14,15]. The tunability of NLC dielectric properties ensures their control by means of small external signals [16]. Finally, the nonlocality of the NLC optical response, *i.e*., with nonlinear perturbation extending much further than the profile of an intense beam itself [17], allows for soliton stabilization in three dimensions, inhibiting catastrophic collapse and filamentation [18]; it also entails long-range interactions between solitons [19,20] and between solitons and external beams [21].

In this paper, we present an overview of spatial solitons in NLC, in short, nematicons, specifically addressing their routing by external electric fields, *i.e*., exploiting the electro-optic response of the medium. In Section 2, we introduce the peculiar optical properties of NLC, detailing how light beams can self-trap through molecular reorientation. We then discuss nematicon propagation in homogeneous samples, outlining their main features and their dependence on both material parameters and geometric configuration. In Section 3, we address the control of nematicon trajectory by means of quasi-static electric fields, illustrating how in-plane and three-dimensional routing can be achieved making use of either index gradients or walk-off variations, respectively, or both of them in synergy.

## 2. Electromagnetic Waves in NLC

Let us start by recalling the basics of linear light propagation in standard NLC. Most NLC are highly birefringent and belong to the class of positive uniaxials [12]. Therefore, their dielectric response is strongly anisotropic at every frequency, with impact on both beams propagation and the response to low-frequency electric fields. Uniaxial NLC have a symmetry or optic axis, often named molecular director *n̂* and microscopically corresponding to the mean alignment of the elongated (rod-like) organic molecules of the mixture. The dielectric constants (refractive indices) associated with field directions (vectors) parallel and perpendicular to the director are often labeled by subscripts, || and ⊥, respectively [13]. In optics, the plane wave eigenvectors are ordinary and extraordinary waves: the first polarized perpendicular to *n̂* and the second with electric field coplanar with both *n̂* and the wavevector ***k***, respectively. Ordinary waves have a phase velocity defined by the refractive index *n_o_*=*n*_⊥_, whereas extraordinary waves depend on a refractive index *n_e_*, which varies with the direction of the wavevector according to $n_{e}={\left({\text{cos}}^{2}\theta /n_{⊥}^{2}+{\text{sin}}^{2}\theta /n_{\|}^{2}\right)}^{-1/2}$, with *θ* the angle of the wavevector with the optic axis *n̂*. Noteworthy, the Poynting vector of an extraordinary wave/beam forms a non-vanishing walk-off angle *δ* with the wavevector ***k***, expressed by $\delta (\theta )=\text{arctan}[{ɛ}_{a}\;\text{sin}(2\theta )/({ɛ}_{a}+2n_{⊥}^{2}+{ɛ}_{a}\;\text{cos}(2\theta ))]$, having introduced the optical anisotropy ${ɛ}_{a}=\left(n_{\|}^{2}-n_{⊥}^{2}\right)$.

**Figure 1:**
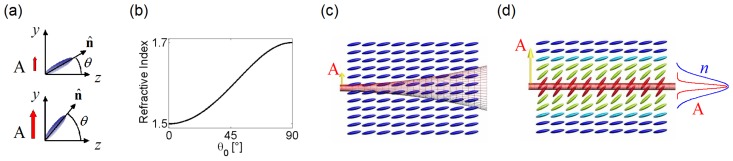
(**a**) Field-dependent reorientation of elongated molecules in positive uniaxial nematic liquid crystals (NLC); (**b**) Extraordinary refractive index *n_e_versus* angle *θ* between *n̂* and ***k*** with reference to the commercial NLC mixture E7; (**c**) Diffraction of a weak light beam in NLC (power is low enough that no appreciable reorientation takes place); (**d**) Intense light beam undergoing self-confinement. The self-focusing reorientational nonlinearity compensates diffraction, and a nematicon is generated. The highly nonlocal response results in an index profile (blue line labeled *n*) much wider than the solitary beam (red line labeled *A*), owing to the intermolecular elastic forces. In panels (**c**,**d**), we ignored the walk-off for simplicity.

We now turn to the nonlinear optical regime, where a specific model is required to describe the (dominant) light-matter interaction. Hereby, we address molecular reorientation in uniaxial NLC. As briefly discussed above, NLC reorientation results from the non-resonant molecular interaction with electromagnetic fields, at either low or optical frequencies, whereby the induced dipoles coplanar with the electric field tend to align their long axis (director) to the field vector (in the case of positive anisotropy *ε_||_> ε*_⊥_), as sketched in Figure 1a. The elastic forces (intermolecular links) in the fluid counteract such electromagnetic torque. The distortion of the director distribution at equilibrium is then determined by a vanishing net torque on the NLC molecules [13]:

(1) $$K\nabla^{2}\theta +\frac{{ɛ}_{0}\Delta ɛ}{2}\;\text{sin }(2\theta )|E{|}^{2}+\frac{{ɛ}_{0}{ɛ}_{a}}{4}\;\text{sin}\;[2(\theta -\delta (\theta ))]|A{|}^{2}=0$$

where *K* is the scalar Frank constant (actually a tensor), *θ* is the reorientation angle due to both low frequency and optical electric fields of amplitude *E* and *A*, respectively, and Δ*ε* = *ε_||_*−*ε*_⊥_ is the dielectric anisotropy. In Equation (1), the first, second and third terms correspond to the elastic, low-frequency electric and optical torques, respectively. In writing Equation (1), we assumed copolarized low frequency and optical electric fields, so that director rotation takes place in one single (principal) plane.

### 2.1. Physics of Nematicon Generation

Reorientation in NLC is strongly polarization-dependent. When the electric field of the light beam is extraordinarily polarized with field and optic axis non-orthogonal to one another, reorientation does not experience the Freedericksz threshold [13], and the director distribution can be modified at every level of optical excitation. When light-induced reorientation becomes appreciable, the distribution of the refractive index changes consistently with the change in *θ*, according to Figure 1b: in a positive uniaxial NLC, the stronger the light intensity *|A|*^2^ is, the higher the extraordinary refractive index becomes. This nonlinear mechanism is the dominant one in undoped NLC subject to extraordinary-wave illumination, in the absence of the Freedericksz threshold and below the transition temperature [13].

Let us focus on a bell-shaped light beam propagating in positive uniaxial NLC with the electric field in the extraordinary polarization. At low power, *i.e*., in the linear optics regime, reorientation is negligible and the beam diffracts (see Figure 1c). When the excitation is high enough to induce an appreciable director rotation, the beam induces a lens-like refractive index distortion, eventually balancing diffraction and supporting self-confinement at appropriate input powers and profiles (see Figure 1d). The simplest self-trapped nonlinear beam in NLC is known as the “nematicon” [15]. Figure 1d illustrates a nematicon and emphasizes one of its fundamental properties, that is, the high degree of spatial nonlocality [18]; owing to the elastic forces between the constituent molecules (the elastic term in Equation (1)), the director perturbation (*i.e*., the index change) extends transversely well beyond the beam size, preventing catastrophic collapse (which occurs in local Kerr media [2,4]) and forming a graded-index waveguide with large numerical aperture [22].

### 2.2. Nematicons in Homogeneous Samples

**Figure 2:**
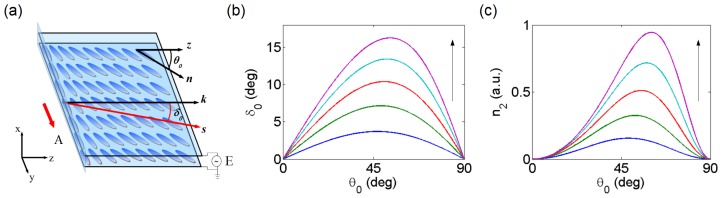
(**a**) Sketch of a bias-free planar NLC cell. The angle *θ*_0_ defines the birefringent walk-off *δ*_0_ = *δ*(*θ*_0_) between the Poynting vector **s** and the wavevector **k**//*ẑ*; (**b**) Walk-off and (**c**) nonlinear (Kerr-like) coefficient versus *θ*_0_ for various degrees of birefringence and *n*_⊥_ = 1.5 (the arrows indicate increasing *n_||_* from 1.6 to 2 in steps of 0.1).

With reference to Figure 2a, we consider a layer of NLC confined between two parallel glass plates, separated by a distance, *h*. Polymer layers (or inorganic films) are deposited on the glass at the interfaces with the NLC and are treated in order to induce the uniform planar alignment (that is, *n̂* · *○* = 0) of the NLC director in the absence of external excitation, at an angle *θ*_0_ with respect to the *z*-axis. Thin films of indium tin oxide (ITO) or other transparent conductors can also be deposited on the inner surfaces, allowing for the application of bias across the NLC. The input beam is usually launched (unless otherwise noted) with a wavevector along the *z*-axis. Two additional glass slides, orthogonal to *ẑ* and rubbed along *y*, seal the sample and avoid material leakage and meniscus formation (hence, undesired light depolarization) at the input [14].

In the paraxial regime and for **k**//*ẑ*, nonlinear propagation of the extraordinary beam (polarized along *y* in the sketch of Figure 2a) can be modeled by a nonlinear Schrödinger-like equation (NLSE) [23]:

(2) $$2ik_{0}n_{e}(\theta_{0})\;\left(\frac{\partial A}{\partial z}+\text{tan }\delta \frac{\partial A}{\partial y}\right)+D_{y}\frac{\partial^{2}A}{\partial y^{2}}+\frac{\partial^{2}A}{\partial x^{2}}+k_{0}^{2}\Delta n_{e}^{2}A=0$$

where *k*_0_ is the vacuum wavenumber, *A* is the (electric) field envelope of the light beam, *D_y_* is the diffraction coefficient across *y* and $\Delta n_{e}^{2}=n_{e}^{2}(\theta )-n_{e}^{2}(\theta_{0})$ is the nonlinear change in the (extraordinary) refractive index. The latter term works as a *photonic* potential and can also account for the role of dielectric inhomogeneities of either linear or nonlinear origins. Using Equation (1), the strength of reorientational self-focusing can be quantified by an equivalent nonlinear Kerr coefficient [16]:

(3) $$n_{2}(\theta_{0})=2\gamma \;\text{sin }[2\;(\theta_{0}-\delta_{0})]\;n_{e}^{2}(\theta_{0})\;\text{tan }\delta_{0}$$

with *γ* = *ε*_0_*ε_a_*/(4*K*). Therefore, both the size of the nonlinearity (proportional to *n*_2_) and the nematicon trajectory (determined by *δ*_0_) can be managed in NLC by controlling *θ*_0_. Figure 2b,c shows the dependence of the walk-off angle *δ* and the figure *n*_2_ on the rest angle *θ*_0_, respectively: both walk-off and nonlinearity are maximized for values of *θ*_0_ slightly above *π*/4. As the birefringence (linked to *ε_a_*) gets larger, both *n*_2_ and *δ* increase, with a more pronounced asymmetry with respect to *π*/4 as *ε_a_* gets larger.

**Figure 3:**
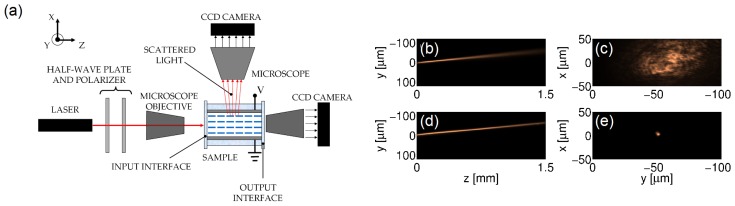
(**a**) Standard experimental set-up. The polarized beam is launched in the sample using a microscope objective. Two CCD cameras allow acquiring of the intensity distribution along the sample in *yz* and at the output in *xy*; Typical images of beam evolution for excitations (**b**) 0.5 mW and (**d**) 2 mW, respectively; (**c** and **e**) corresponding output profiles after 1.5 mm propagation.

Figure 3 shows a standard experimental set-up and typical acquired images of the evolution and output profile of an extraordinarily polarized beam of wavelength *λ* = 1064 nm launched in the mid-plane of an *h* = 75 μm planar cell with E7. The input beam is focused with a waist *w*_0_ ≈ 3 μm. The director at rest is homogeneously aligned at *θ*_0_ = 45º. A CCD (Charge Coupled Device) camera allows collecting of the scattered light from above the cell and observing the beam propagation in the plane, *yz*; another CCD camera acquires the output profile in *xy*. We stress that the acquired images of beam propagation (*yz* plane) are a blurred replica of the actual beam due to light scattering in liquid crystalline materials [24]. At low power (Figure 3b,c), the reorientation is negligible, and the beam diffracts, while at moderate powers (Figure 3d,e), the optically-induced refractive index increase balances diffraction and confines light into a nematicon, according to Equations (1) and (2). The large birefringence, on the order of 0.2 in E7, makes both linear and soliton beams propagate at an angle *δ* ≈ 7º with respect to the wavevector ***k***[25].

**Figure 4:**
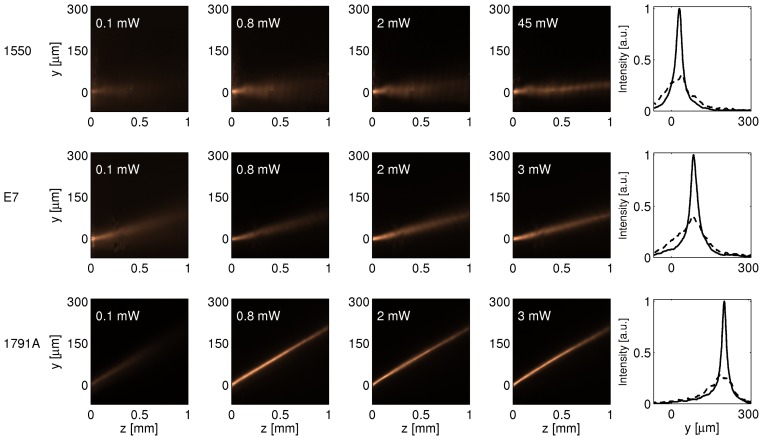
Beam propagation in three (undoped) NLC mixtures. As birefringence goes up, walk-off increases, whereas self-confinement is appreciable at lower powers. The rightmost graphs plot linear (dashed line) and nonlinear (solid line, corresponding to the highest excitation) output beam profiles across *y*.

Experimental observations with various NLC mixtures, carried out to underline the role of material parameters, confirmed the theoretical predictions: Figure 4 shows the images acquired from three identical samples filled with distinct NLC, namely 1550, E7 and 1791A [26]. At *λ* = 1064 nm, they exhibit Δ*n* = *n_||_* −*n*_⊥_ = 0.05*,* 0.2 and 0.4, respectively, all with *n*_⊥_ ≈ 1.5; consistently, the corresponding measured walk-off angles were *δ* = 2.5º, 6.5º and 12º, respectively. The input powers required for self-trapping were *P* = 45, 2 and 0.8 mW, respectively, *i.e*., the larger *n*_2_ is, the lower the excitation needed for nematicon formation.

## 3. Controlling Nematicon Trajectory with External Bias

As mentioned in the Introduction, the control of nematicon trajectory is essential for their use as readdressable waveguides in reconfigurable interconnects and switching circuits for optical signal processing. Nematicons can be steered in direction by two main approaches: modifying the walk-off or introducing index gradients in the medium, respectively. The walk-off *δ* of a nematicon can be changed by acting on the *average* director orientation: for example, with reference to Figure 2a, by varying *θ*_0_, the direction of propagation (Poynting vector) would change according to Figure 2b. Noteworthy, with this approach, the soliton wavevector (*i.e*., phase wavefront) remains unaltered. Alternatively, the nematicon path can be modified by inducing non-uniform director distributions (*i.e*., *θ*_0_ depending on the spatial coordinates), thus creating index gradients able to deflect the self-guided wave packet according to the refractive index landscape in which it propagates.

To give a mathematical basis to these physical statements, let us refer to Equation (2): since the propagation of an optical spatial soliton is formally equivalent to the motion of a charged particle in an electromagnetic field, we can recast the Ehrenfest theorem and obtain the mean equivalent force, *F*, acting on the beam: [16]:

(4) $$F\approx {\frac{1}{n_{e}(\theta_{b})}\nabla_{xy}n_{e}(\theta )|}_{r_{b}}+\frac{\partial \;\text{tan }\delta_{b}}{\partial z}\hat{y}$$

where the subscript, *b*, refers to a quantity computed on the beam axis and ▽*_xy_* = *○*∂/∂*x* + *ŷ*∂/∂*y*. As anticipated, Equation (4) states that the nematicon trajectory depends on both the transverse refractive index gradients (the first term on the RHS) and the longitudinal variations of the walk-off (second term). Having laid out the basics of nematicon propagation in an inhomogeneous NLC environment, hereafter, we illustrate a few strategies to control nematicon direction using an external bias and exploiting electro-optic reorientation (see Section 2).

### 3.1. Three Dimensional Steering

Nematicons are beams that propagate according to input wavevector and polarization, as well as to birefringent walk-off and medium inhomogeneities (if present). Therefore, with the exception of specific launch conditions in uniform samples, in general, their energy flow in the NLC volume has a three-dimensional character [27].

#### 3.1.1. Planar Uniform Electrodes

**Figure 5:**
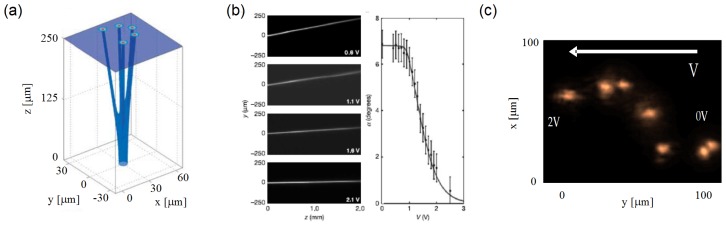
(**a**) Sketch of 3D beam steering via a uniform electric field applied across the NLC thickness (*i.e*., along *x*); only walk-off changes are accounted for; (**b**) Photographs of nematicons propagating at different biases and a graph of corresponding apparent walk-off in the plane *yz* (dots with error bars are experimental data; the solid line is the theoretical prediction); (**c**) Superposition of the output beam profiles (after 1-mm propagation) for various applied voltages; these results also reveal the interaction of the nematicon with the cell boundaries (*h* = 100 μm). The white arrow indicates an increasing bias. Here, the used wavelength was 1064 nm, and the input wavevector was parallel to *ẑ*.

Let us consider the planar cell described in Figure 2a with *θ*_0_ = 45º and *h* = 100 μm, filled with the nematic E7 (Δ*n* ≈ 0.2 at *λ* = 1064 nm, the employed laser wavelength). By rotating the polarization of the incoming beam, it is possible to couple all its power into ordinary or extraordinary wave components [28]. Ordinary beams diffract, as the electric field is orthogonal to the optic axis and the Freedericksz threshold prevents reorientation (the previous statement is strictly valid at moderate powers at which thermal effects can be ignored). Extraordinary beams undergo self-focusing, eventually forming nematicons at high enough excitations [29], with properties strongly depending on the applied voltage. In fact, in the unbiased case, nematicons lie in the plane, *yz*, and propagate with a walk-off angle *δ* ≈ 7º with respect to *z*. An external voltage with field lines along *x* can move the NLC molecules (*i.e*., the director) out of the *yz* plane, varying the orientation of the optic axis, *i.e*., altering its angle with the soliton wavevector and the resulting nematicon walk-off (Figure 5a).

Figure 5b shows the observed beam evolution in the plane *yz* (set-up as in Figure 3a) [29]. Viewing the cell from above (along *x*) allows measuring of the “apparent” walk-off *α* (Figure 5b), *i.e*., the angle between Poynting and wave-vectors as it appears in the observation plane *yz*. The measurement of the output profile (Figure 5c) indicates a more complicated dynamics than in Figure 5a. In fact, the bias- driven reorientation induces a one-dimensional waveguide able to confine light along *x* and as thick as the cell itself, thus with a multimodal character. The nematicon moves in this graded-index environment, which can alter its wavevector ***k***. Thus, both walk-off and wavevector changes coexist, as confirmed by numerical simulations and experimental studies of the nematicon trajectory also in the plane *xz* [27]. Moreover, NLC properties, such as nonlinearity and nonlocality, depend on the applied bias [30]. Finally, we note that, in this configuration, the maximum steering in the plane *yz* depends on the available walk-off, *i.e*., on the medium birefringence (see Figure 2b), with *α* spanning from 7º to 0º when using E7 (see Figures 2b and 5b).

#### 3.1.2. Tailored Electrodes

**Figure 6:**
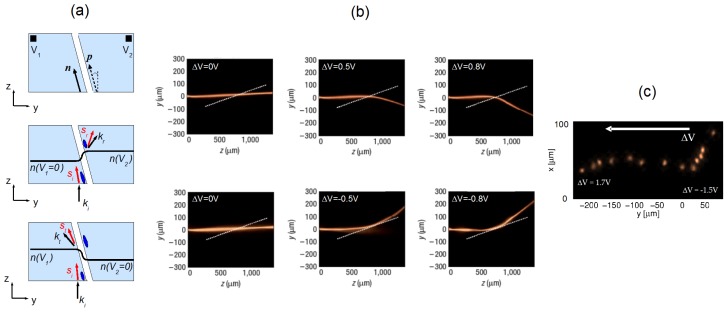
(**a**) Sketch of the sample with an electrically defined interface and interaction geometry: subscripts *i*, *r* and *t* label incident, refracted and reflected Poynting vectors (***s***) and wavevectors (***k***), respectively; (**b**) Acquired images of refraction (**top panels**) and total internal reflection (TIR) (**bottom**) for various Δ*V* ; (**c**) Superposed photos of output beam profiles after 1 mm propagation; the white arrow indicates an increasing Δ*V*.

The achievable nematicon deflection in a planar cell can be increased in geometries that maximize the refractive index gradients: in this case, the steering is mainly due to wavevector variations and depends on both the interval spanned by *n_e_* (upper bound Δ*n*) and on the configuration. The largest voltage-tunable 3D deflection was achieved introducing a gap in one of the planar electrodes, *i.e*., dividing it into two as sketched in Figure 6a[31]. The upper conducting ITO film was split into two regions (namely 1 and 2), with a straight gap (about 100 μm-wide) along the direction, *p*, at ≈ 10º with respect to *z*. This allowed the application of two independent voltages, *V*_1_ and *V*_2_, in regions 1 (on the left in Figure 6a) and 2 (on the right), respectively, so that a non-uniform director distribution was impressed in the NLC layer (see Figure 6a): the net effect was the formation of a dielectric interface across the gap, with a change in optic axis orientation between the two biased regions. With the bottom (ground) electrode in common, the graded-index interface could be tuned by adjusting *V*_1_ and *V*_2_ in sign and/or magnitude.

We stress that, due to the adiabaticity of the graded director distribution, in this geometry, light propagates in the Mauguin limit with no power coupling between ordinary and extraordinary components [28], preventing beam splitting at the interface [31]. Since nematicons are extraordinarily polarized beams, a director tilt out of the plane *yz* corresponds to a net increase in the refractive index. Thus, when Δ*V* = *V*_2_*–V*_1_*>* 0, the refractive index experienced by the propagating soliton is higher in region 2, *i.e*., past the interface along *z*: a nematicon travels across and undergoes refraction (Figure 6b, top panels). The overall mismatch in refractive index increases with net bias; thus, the deflection of both the Poynting vector (***s****_r_*) and the wavevector (***k****_r_*) increases with the voltage difference *|*Δ*V |*. Conversely, when Δ*V <* 0, the refractive index is larger in region 1 and total internal reflection (TIR) of the nematicon can occur above critical angle incidence (Figure 6b, bottom panels). Noticeably, the beam maintains its self-trapped character even after interacting with the interface, demonstrating its robustness and stability to external perturbations. With this geometry, an overall steering angle of about 40º could be achieved, going from refraction to TIR as Δ*V* was tuned. However, even in this configuration, due to out-of plane molecular reorientation, the principal plane becomes voltage-dependent, moving the soliton in a 3D trajectory as illustrated in Section 3.1.1. Typical output profiles of nematicons are displayed in Figure 6c[32].

### 3.2. In-Plane Steering

**Figure 7:**
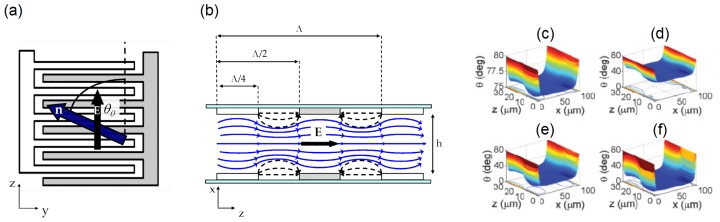
(**a**) Electrode configuration. The main component of the electric field is along *z*, thus inducing director rotation towards *z* and coplanar with *yz*; (**b**) Side view. The black dashed lines represent the electric field lines. The solid blue lines indicate the director distribution inside the sample, with no *x* component in the mid-plane; (**c**–**f**) Numerically calculated reorientation for (**c**) *V* = 1; (**d**) 2; (**e**) 3 and (**f**) 5 V, respectively, showing a flat director profile (*θ*) in the cell core *x* ≈ *h*/2.

The 3D motion of nematicons discussed in Section 3.1 is quite sensitive to the launch conditions, such as spurious wavevector tilt and misalignment with respect to the cell mid-plane. More reliable control of the nematicon trajectory can be obtained in geometries ensuring director rotation within the propagation plane. To this extent, the electrodes need to be designed so that the applied electric field reorients *n̂* in *yz*. A sample arrangement is illustrated in Figure 7a,b [33]: top and bottom conductive (ITO) electrodes are defined into two interdigitated comb-like structures with fingers along *ŷ*, the width of each being Λ/4 = 15 μm. The planar cell is *h* = 100 μm-thick, and the director is aligned at 80º with the *z*-axis at rest, in order to maximize the steering range achievable by the application of a voltage. When the electrodes are biased, an electric field distribution of period Λ is established in the region close to each glass/NLC interface, determining a periodic molecular reorientation of period Λ/2. Due to the relative sizes, the condition Λ/4 *<< h* guarantees that the dominant electric field is directed along *z*, and the resulting electric torque moves the NLC molecules essentially within the plane *yz*. The elastic (intermolecular) forces “diffuse” the bias-driven reorientation towards the cell core (*x* ≈ *h*/2), with an additional smoothing of the rapid field variations along *y* through the nonlocal response [16]. To a good approximation, in the mid-plane *x* = *h*/2, reorientation occurs solely in the *yz* plane, where the solitary beam is launched and propagates, as drawn in Figure 7b. Numerical simulations (Figure 7c–f) confirm that the combination of electro-optic and elastic responses yields in-plane director reorientation: the cell with interdigitated electrodes effectively behaves as a standard planar cell (as the one plotted in Figure 2a), but with molecular alignment at an angle tuned by the applied voltage, *V*, *i.e*., with *θ*_0_ = *θ*_0_(*V* ).

**Figure 8:**
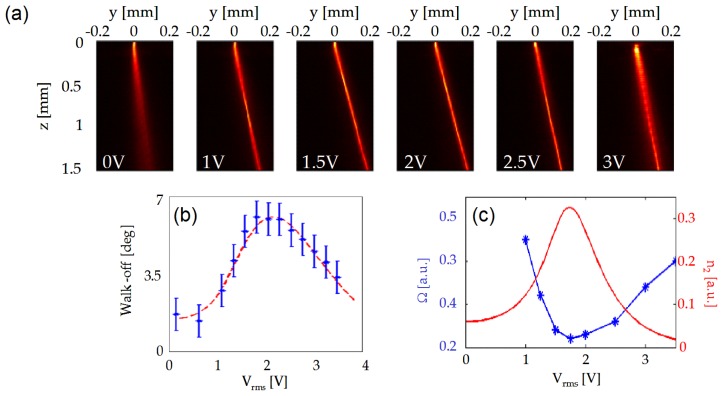
(**a**) Photographs of *λ* = 1064 nm nematicons steered in a sample with interdigitated electrodes as in Figure 7 for various applied voltages, *V* ; (**b**) Measured (blue dots) and theoretical (red dashed line) walk-off *versus* bias; (**c**) Comparison between the equivalent nonlinear coefficient *n*_2_ (red solid line) and the nematicon breathing period Ω (blue stars) *versus* applied voltage.

Figure 8a presents photographs of a *P* = 2 mW beam launched in the cell mid-plane *x* = *h*/2 for various applied voltages *V*. The beam deflection can be entirely ascribed to the induced variations in walk-off; moreover, the angle measured in the *yz* plane is the actual walk-off rather than its apparent value, at variance with the case of uniform electrodes. Walk-off, and, thus, the beam steering angle, spans from *δ* ≈ 2.5º, corresponding to *θ*_0_ = *θ*_0_ (*V* = 0 V), to the maximum available *δ* ≈ 7º, achieved for *θ*_0_ = *θ*_0_(*V* = 2.5 V) ≈ 45º (see Figure 2b); for even larger *V*, the walk-off starts to decrease as expected; see Figure 8b. The largest applied voltage used (*V* = 4 V) is limited by the insurgence of non-negligible *x* components of the electric field.

Analyzing the beam evolution in the NLC, one can notice that also the magnitude of the nonlinearity changes, with stronger self-focusing for a given excitation. In fact, by varying *θ*_0_, the effective nonlinear coefficient obeys Equation (3): without bias, the nonlinearity (plotted in Figure 8c*versus V* ) is too low to allow solitonic propagation and the beam diffracts; increasing *V*, the beam undergoes self-confinement, with a breathing period Ω [24] getting shorter for higher nonlinearity (see Figure 8c), as expected in nonlocal Kerr-like media [16,17].

#### 3.2.1. In-Plane Refraction and TIR at Graded Interfaces

**Figure 9:**
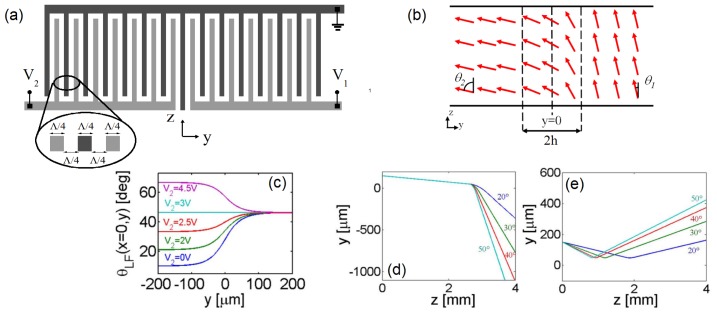
(**a**) Electrode geometry and (**b**) operation of a sample with an in-plane electrically-defined graded interface; (**c**) Calculated director distribution in the cell mid-plane *x* = *h*/2 *versus y* for *V*_1_ = 3 V and several *V*_2_. The trajectory of a soliton undergoing (**d**) refraction (*θ*_2_*> θ*_1_, with *θ*_1_ = 10º ) and (**e**) TIR (*θ*_1_*> θ*_2_, with *θ*_2_ = 10º ); in panels (**d**,**e**), the corresponding values assumed by *θ*_2_ are indicated next to each line. The interface is located in *y* = 0.

A natural extension of what is discussed in Section 3.2 is the use of interdigitated electrodes to define in-plane dielectric interfaces, analogously to Section 3.1.2. ; an in-plane interface is expected to maximize the angular steering via wavevector changes through an index gradient. Let us examine the geometry in Figure 9: on each glass slide of the planar cell, two comb-like interdigitated electrodes are realized with the same size as in Section 3.2, but with fingers along *ẑ*; the common ground electrode also separates the two independently biased regions. In the absence of applied voltage(s), the director is aligned at an angle of 10º with *z*. As confirmed by numerical simulations using Equation (1) (with *A* = 0, *i.e*., in the absence of light), when distinct voltages are applied to the combs, the periodic electric field distribution defines two NLC regions with planar director orientations (Figure 9c), with an intermediate transition region where the director orientation evolves almost linearly for ≈ 2*h*: thus, the sample operates as a voltage-controlled graded dielectric interface parallel to *ẑ* [34] and in-plane (*yz*) director distribution. The nematicon trajectories for input wavevectors ***k****|| ẑ* were computed via Equation (4) and are shown in Figure 9d,e, assuming the input beam is launched in region 1 corresponding to *y >* 0 (in region 2 *y <* 0). We note that, due to the initial walk-off, nematicons interact with the interface, even when the wavevector, ***k***, is parallel to *ẑ*, allowing larger overall deflections as compared to an isotropic material [35]. The interface attracts and repels the soliton when *V*_1_*< V*_2_ and *V*_1_*> V*_2_, respectively, with the output nematicon position and angle depending on both the bias difference Δ*V* = *V*_1_*–V*_2_ and the absolute value *|V*_1_*|* (or *|V*_2_*|*), due to the medium anisotropy [35].

**Figure 10:**
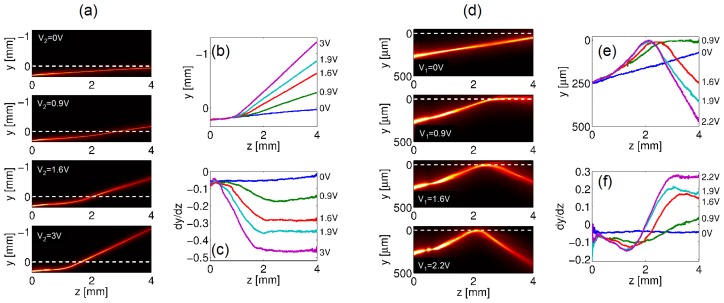
(**a**) Photographs of nematicon refraction at an interface defined by various values of *V*_2_ when *V*_1_ = 0; Corresponding graphs of (**b**) nematicon trajectory *y* = *y*(*z*) and (**c**) its first derivative; (**d**) Photographs of nematicon total internal reflection at the graded interface, for various *V*_1_, when *V*_2_ = 0. Corresponding graphs of (**e**) nematicon trajectory in *yz* and (**f**) its slope. Noteworthy, non-specular reflection can be observed, due to the optical anisotropy [35,36].

In the experiments, we launched a TEM_00_ beam of waist *w*_0_ ≈ 3 μm from region 1 (*y >* 0) with ***k****||ẑ* and *P* = 5 mW, the latter ensuring nematicon formation regardless of the actual *V*_1_. Figure 10 shows the observed propagation and deflection of nematicons. For *V*_1_ = 0, the refractive index in region 2 is higher than in region 1: the nematicon is refracted in region 2 going through the interface. Increasing *V*_2_, the angle of refraction increases (Figure 10a–c). Conversely, when *V*_2_ = 0, the angle of incidence changes with *V*_1_, due to walk-off; in this case, the Poynting vector after the transition can point towards negative *y*, due to significant walk-off, resulting in nematicon transmission through the interface, even under TIR conditions for the wavevector; for instance, Figure 10d (*V*_1_ = 0.9 V) displays an outgoing nematicon that propagates nearly parallel to the interface [35]. For *V*_1_*>* 0.9 V, the soliton undergoes TIR; see Figure 10e,f.

**Figure 11:**
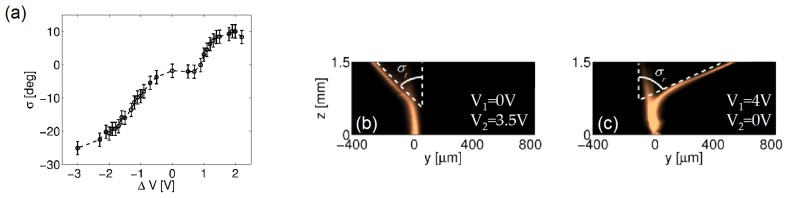
(**a**) Overall deflection obtained with a nematicon at grazing incidence *versus* bias difference Δ*V* = *V*_1_*–V*_2_, with *V*_1_ (*V*_2_) vanishing when *V*_1_*< V*_2_ (*V*_1_*> V*_2_); (**b**) Refracted and (**c**) totally internally reflected nematicon with an angle of incidence of ≈ 16º with *ẑ*; In (**c**), some losses are appreciable, owing to the abruptness of the dielectric barrier. This launch condition maximizes the deflection, reaching *σ* ≈ 55º.

The overall deflection *σ*, plotted in Figure 11a for −3V *<* Δ*V <* 2V (*i.e*., including transmission and total internal reflection), was about 35º. Such an angular range can be increased by tilting the input beam (wavevector), as shown in Figure 11b,c: for an input angle of ≈ 16º, we achieved an overall deflection (refraction angle *σ_t_* plus reflection angle *σ_r_*) of about ≈ 55º, the largest nematicon steering to date in an electro-optically controlled arrangement [37]. Larger soliton deviations, reported at air-NLC interfaces, lacked tunability and/or voltage control [38,39]).

### 3.3. Deflection Controlled by the Applied Frequency

In the previous sections, we reviewed configurations that permit us to control the nematicon direction by varying the amplitude of the applied bias *V*. In specific NLC compounds, however, it is also possible to control the soliton trajectory by simply varying the frequency of the applied quasi-static electric field. In standard undoped NLC, the dielectric anisotropy Δ*ε* depends slightly on the frequency up to the MHz region. In this interval, it decreases until vanishing at a crossover frequency, *f_c_*, before eventually changing sign: thus, the torque ***M*** becomes strongly dependent on frequency, *i.e*., ***M*** = *ε*_0_Δ*ε*(*f*) *|E|*^2^. In the nematic mixtures, named Double Frequency Liquid Crystals (DFLC), the crossover frequency lies in the lower range, 1–10 kHz [40], offering the possibility to modulate the director orientation by varying the frequency (not only the magnitude) of the applied voltage.

**Figure 12:**
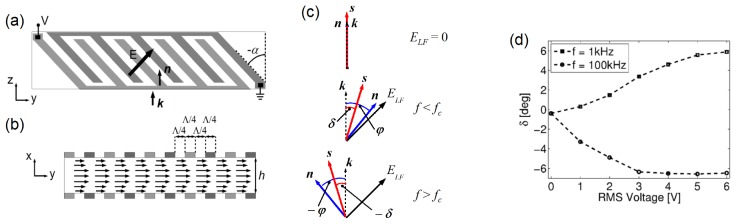
(**a**) Top and (**b**) side view of the planar cell with tailored interdigitated electrodes. The applied electric field forms an angle of 45º with respect to the initial director *n̂ ||ẑ*; (**c**) Sketch of the electrically-driven reorientation: the director *n̂* and the Poynting vector ***s*** tend to reorient towards (positive anisotropy) or away from (negative anisotropy) the electric field vector at frequencies below or above the crossover value, *f_c_*, respectively; (**d**) Plot of the measured walk-off *versus* voltage for positive (*f* = 1 kHz, squares) and negative (*f* = 100 kHz, dots) NLC anisotropies and director rotations, respectively.

To exploit this effect we designed a planar cell containing the DFLC MLC-2048, as drawn in Figure 12a,b; interdigitated comb electrodes were deposited on each of the confining glass plates, with Λ *< h*, to ensure planar director reorientation in the mid-plane, as described in Section 3.2. In order to maximize the steering angle, the finger direction was set at 45º with respect to *z*, whereas the director alignment at rest was along *ẑ*. Due to the electrode configuration, an applied voltage established an electric field at 45º with respect to *ẑ*, resulting in a steady (uniform) director distribution at +45º or −45º for frequencies lower or higher than *f_c_*, respectively, as sketched in Figure 12c. The outcome was the frequency-controlled rotation of the optic axis in the interval [−*π*/2 *π*/2] for large enough biases [41].

A change in the frequency of the applied electric field could affect both transverse confinement and trajectory of the nematicons, as both the nonlinear coefficient *n*_2_ and the walk-off depend on the angle between the director and the beam wavevector (see Figure 2b,c). As summarized in Figure 12d, when the bias frequency was *f* = 1 kHz, the nematicon path changed with the amplitude *V* as in standard NLC, with a positive walk-off reaching its maximum *δ* = *δ_MAX_* ≈ 6.5º for *V* ≈ 6 V. Conversely, when *f* = 100 kHz, the walk-off was negative, until it reached the saturation value *δ* = −*δ_MAX_* for *V* ≈ 3 V.

**Figure 13:**
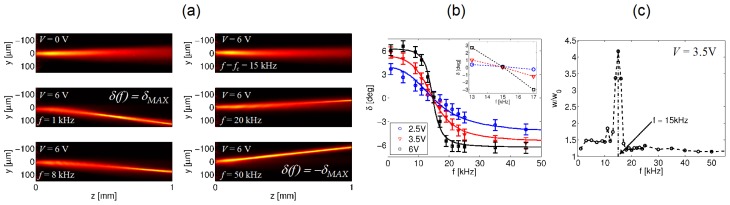
(**a**) Photographs of frequency-controlled nematicon steering; (**b**)Walk-off *versus* bias frequency for various *rms* (root mean square) voltage values. The lines intersect when, at the crossover frequency, the dielectric anisotropy is zero and, therefore, there is no electric reorientation; (**c**) Relative beam width (*w*_0_ is the initial waist) *versus* frequency. The frequency-dependent reorientation also affects the nonlinearity, which vanishes at the crossover value.

For *V* = 2.5, 3.5 and 6 V, we varied the frequency, *f*, from *f* = 1 kHz to *f* = 100 kHz and measured the soliton walk-off *δ*(*f*), as graphed in Figure 13b. Starting from the value at low frequency, the walk-off decreased until 0º at *f* = *f_c_* for all three voltages; then, it changed sign for *f > f_c_*, *i.e*., when the electric torque changed sign. Moreover, as is visible from the acquired images in Figure 13a and from the graph of the waist *versus f* in Figure 13c, the nonlinear response strongly depended on *f*, with the beam loosing confinement for *f* = *f_c_*, where the anisotropy, and thus, *n*_2_, according to Equation (3), became negligible. Using this dependence of the optic axis reorientation from frequency, we could maximize the steering exclusively due to walk-off variations up to Δ*δ* ≈ 13º, with a deflection twice larger than in standard NLC (see Figure 8).

## 4. Conclusions

We have shown how all-optical reorientation in nematic liquid crystals sustains self-focusing and the formation of stable optical spatial solitons, stressing that material and geometric properties affect the nematicon propagation, including trajectory and width. Based on the electro-optic response of NLC, we have reported and discussed various strategies to achieve and maximize the voltage-controlled addressing of nematicons, *i.e*., self-confined light beams and the associated light-induced waveguides. Owing to the large dielectric anisotropy of NLC, we have illustrated how the nematicon path can be changed through longitudinal variations in walk-off and transverse gradients in index. While walk-off variations yield, in general, smaller deflections than index gradients, the best approach depends on the specific configuration. Both 3D and in-plane voltage-tunable nematicon deflections can be engineered, controlled by either the amplitude(s) or the frequency of the external bias.

While spatial optical solitons in nematic liquid crystals form an ideal platform for the development of a new generations of photonic guided-wave networks for all-optical signal processing, switching and routing, the wealth of the reported approaches for the controlled steering of nematicons lets us envision further developments in neighboring areas of optics. Among them, we like to mention their use as intense optical probes for the physical characterization (thermal, dielectric and elastic properties) of new mixtures of liquid crystals, including the complex interaction between NLC (host) and dopant (guest) molecules [13] and the investigation of high-field photochemical reactions; their role in active guided-wave optics (e.g., for amplification, lasing); and their operation as optical tweezers to control the position of micro- and nano-particles [42]. Finally, nematicons and their high photon densities could help in studying the transitions between different phases of (soft) matter and the reciprocal interaction between photons and matter in highly nonlinear regimes [43].
